# RHOG Activates RAC1 through CDC42 Leading to Tube Formation in Vascular Endothelial Cells

**DOI:** 10.3390/cells8020171

**Published:** 2019-02-18

**Authors:** Oula El Atat, Amira Fakih, Mirvat El-Sibai

**Affiliations:** Department of Natural Sciences, Lebanese American University, Beirut 1102 2801, Lebanon; oula.elatat@lau.edu.lb (O.E.A.); amira.fakih@lau.edu.lb (A.F.)

**Keywords:** RHOG, RAC1, CDC42, RHO GTPases, angiogenesis, vascular endothelial cells

## Abstract

Angiogenesis is a hallmark of cancer cell malignancy. The role of the RHO family GTPase RHOG in angiogenesis in vascular endothelial cells has recently been elucidated. However, the regulation of RHOG during this process, as well as its cross-talk with other RHO GTPases, have yet to be fully examined. In this study, we found that siRNA-mediated depletion of RHOG strongly inhibits tube formation in vascular endothelial cells (ECV cells), an effect reversed by transfecting dominant active constructs of CDC42 or RAC1 in the RHOG-depleted cells. We also found CDC42 to be upstream from RAC1 in these cells. Inhibiting either Phosphatidyl inositol (3) kinase (PI3K) with Wortmannin or the mitogen-activated protein kinase extracellular-regulated kinase (MAPK ERK) with U0126 leads to the inhibition of tube formation. While knocking down either RHO, GTPase did not affect p-AKT levels, and p-ERK decreased in response to the knocking down of RHOG, CDC42 or RAC1. Recovering active RHO GTPases in U0126-treated cells also did not reverse the inhibition of tube formation, placing ERK downstream from PI3K-RHOG-CDC42-RAC1 in vascular endothelial cells. Finally, RHOA and the Rho activated protein kinases ROCK1 and ROCK2 positively regulated tube formation independently of ERK, while RHOC seemed to inhibit the process. Collectively, our data confirmed the essential role of RHOG in angiogenesis, shedding light on a potential new therapeutic target for cancer malignancy and metastasis.

## 1. Introduction

Angiogenesis is the formation of new blood vessels from preexisting blood vessels [1]. It is required during embryonic development, growth, regeneration and wound healing [1,2]. Furthermore, it is implicated in pathological processes including arthritis, muscular dystrophy, and tumorigenesis [3]. During tumorigenesis, the angiogenic process starts when endothelial cells (EC) branch from pre-existing small vessels, forming sprouts of capillaries that can supply the tissue of the tumor [2]. The new blood vessel formation is triggered by angiogenic stimuli, such as the vascular endothelial growth factor (VEGF) [1,4]. Growth factors stimulate the proliferation and migration of ECs through the activation of the phosphatidylinositol (3) kinase (PI3K) pathway as well as the Mitogen-activated protein kinase (MAPK) pathway [5,6,7]. New blood vessel formation is also regulated by the RHO GTPase family [8,9,10].

The RHO family of GTPases is comprised of 20 members of small GTP binding proteins with molecular sizes ranging from 20–40 KDa [11]. The most considered RHO GTPases are RHOA/C, RAC1, and CDC42 [12,13,14]. RHO GTPases act as switches between active GTP-binding states and inactive GDP-binding states [11]. They are activated by the guanine nucleotide exchange factors, (GEFs) which are, in many cell types, activated downstream from PI3K [15,16]. GTPases-activating proteins (GAPs) and Guanine dissociation inhibitors (GDIs) lead to the inactivation of RHO GTPases (Schmidt & Hall, 2002). RHO GTPases are key regulators of the actin cytoskeleton and actin remodeling events required during cellular proliferation and migration [17,18,19]. RHOA, RHOC, RAC1, and CDC42 regulate endothelial cell proliferation, polarization, cell-cell adhesion, and migration, as well as vascular permeability during angiogenesis [8,9,20,21,22,23,24,25].

RHOG is an emerging RHO GTPase that possesses almost the same sequence as RAC1 and CDC42 and that has been found to be critical for the regulation of the actin cytoskeleton and migration in many cell types [26,27,28,29,30,31,32]. In addition, several reports also established a cross-talk between RHOG and RAC1 and CDC42, where RHOG was found to activate CDC42/RAC1 in some systems [28,31,33,34]. To date, the role of RHOG in vascular endothelial cells during angiogenesis has not been elucidated. In this study, we investigated the role of RHOG in tube formation in vascular endothelial cells. We also examined its regulation, cross-talk with other RHO GTPases, and potential downstream effectors. This established RHOG as a potentially promising new target for anti-angiogenesis therapeutic applications in cancer.

## 2. Materials and Methods

### 2.1. Cells and Culture Conditions

Human endothelial cells ECV were cultured in RPMI 1640 supplemented with 10% Fetal Bovine Serum (FBS) and 100 U/µL of penicillin/streptomycin at 37 °C and 5% CO_2_ in a humidified chamber.

### 2.2. Antibodies and Reagents

The following primary antibodies were used in this study: Mouse monoclonal anti-RHOG, mouse monoclonal anti-RHOA, rabbit polyclonal anti-RHOC, mouse monoclonal anti-RAC1, rabbit monoclonal anti-ROCK1, rabbit monoclonal anti-ROCK2, rabbit polyclonal anti-p-AKT1 (phospho-S473), rabbit polyclonal anti-pan-AKT, and rabbit polyclonal anti-Actin antibodies, as well as the MEK1/2 inhibitor U0126 and the PI3K inhibitor Wortmannin, which were obtained from Abcam (Abcam Inc., Cambridge, UK). Rabbit polyclonal anti-CDC42 (sc-87), mouse monoclonal anti-p-ERK1 (phospho-Y204), mouse monoclonal anti-ERK, and goat polyclonal anti-STARD13 antibodies were obtained from Santa Cruz (Santa Cruz Biotechnology., Santa Cruz, CA, US). Anti-rabbit and anti-mouse HRP-conjugated secondary antibodies were obtained from Promega (Promega Co., Maddison, WI, USA). The Dominant active Cherry-tagged RAC1-Q61L and Cherry-tagged CDC42-Q61L constructs were a generous gift from Dr. Louis Hodgson.

### 2.3. Cell Transfection and Small Interfering RNA

Cells were transfected with 5 μg dominant active RAC1, dominant active CDC42, or the control empty vector (pcDNA3.1) using Lipfectamine LTX with Plus reagent (Invitrogen, Carlsbad, CA, USA) as described by the manufacturer. The experiments were performed 24 h following transfection. Human FlexiTube siRNA for each of RHOG [oligo 1, 2 and 3 out of the NM_001665 panel), RAC1 (oligo 5 and 6 out of the NM_006908, NM_018890 and NM_198829 panels), CDC42 (oligo 4 and 7 out of the NM_001039802, NM_001791 and NM_044472 panels), STARD13 (oligo 4 out of the NM_001243466 panel], RHOA (oligo 1 and 6 out of the NM_001664 panel), RHOC (oligo 5 and 6 out of the NM_001042678, NM_00104269 and NM_175744 panels), ROCK1 (oligo 9 and 10 out of the NM_005406 panel) and ROCK2 (oligo 5 and 6 out of the NM_004850 panel) were brought from Qiagen (Qiagen, Hilden, Germany). The cells were transfected with the siRNA at a final concentration of 10 nM using HiPerfect (Qiagen, Hilden, Germany) as described by the manufacturer. Control cells were transfected with siRNA sequences targeting GL2 Luciferase (Qiagen, Hilden, Germany). After 72 h, the protein levels in the total cell lysates were analyzed by Western blotting using the appropriate antibodies, or the effect of the corresponding knockdown was assayed. Western blot analyses were performed to choose the oligos showing the most effective knockdowns from every FlexiTube panel corresponding to the target sequence of proteins mentioned above. These oligos were used in this study, and Western blots showing knockdown efficiency were also included in the study. Cells transfected with both siRNA and a vector were incubated with the siRNA for 72 h as previously mentioned then co-transfected with the appropriate vector 24 h prior to the experiment.

### 2.4. Immunoblotting

Cell lysates were prepared by scraping the cells with Laemmli Sample Buffer (LSB) containing 4% Sodium Dodecyl Sulfate SDS, 20% Glycerol, 10% β-Mercaptoethanol, 0.004% Bromophenol Blue, and 0.125 M Tris-HCl (pH = 6.8). The resulting lysates were boiled for 5 min. Protein samples were separated by SDS-PAGE gels and transferred to PVDF membranes. Membranes were then blocked either by 5% BSA for 2 h at room temperature depending on the primary antibody used. The membranes were then incubated with the appropriate primary antibody for 2 h. After that, the membranes were washed and incubated with secondary antibodies at a concentration of 1:2000 for 1 h at room temperature. Bands were visualized by treating the membranes with a chemiluminescent reagent Enhanced Chemiluminescence-ECL detection kit (GE Healthcare, Chicago, IL, USA). The levels of expression of proteins were compared by densitometry using the ImageJ software and plotted in Excel.

### 2.5. Pull Down Assays

The pull-down assays were performed using the RHOA/RAC1/CDC42 Activation Assay Combo Kit (Cell BioLabs, San Diego, CA, USA) following the manufacturer’s instructions. Briefly, cell lysates were incubated with GST-RBD (for RHOA pull-down) or GST-CRIB (for RAC1/CDC42 pull-down) for 1 h at 4 °C with gentle agitation. Then, the samples were centrifuged, and the pellet was washed several times. After the last wash, the pellets were resuspended with sample buffer and boiled for 5 min. GTP-RHOA, GTP-RAC1, and GTP-CDC42 were detected by Western blotting using anti-RHOA, anti-RAC1, and anti-CDC42, respectively. Total RHOA/RAC1 and CDC42 were collected prior to the incubation with GST-RBD/GST-CRIB and used as a loading control. All Western blots were analyzed with ECL (GE Healthcare, Chicago, Il, USA) followed by densitometry.

### 2.6. The Tube Formation Assay

Matrigel™ basement membrane matrix (growth factor reduced) (BD Biosciences, San Jose, CA, USA) was thawed at 4 °C, pipetted into pre-cooled 35 mm Petri dishes, and incubated at 37 °C for 1 h. After Matrigel polymeration, treated cells were suspended in RPMI medium and were seeded onto the Matrigel. For tube formation analysis, images were taken using infinity corrected optics on a Nikon Eclipse microscope supplemented with a computer-driven Roper cooled CCD camera and operated by IPLab Spectrum software v. 2.8 (BD Bioscience, San Jose, CA, USA, 2012) after 24, 48, and 72 h using 10× objective. The tube formation assay was analyzed and interpreted using the WimTube software (Wimasis, Munich, Germany, 2015, Image Analysis,) based on different parameters such as branching points, tube lengths, tube numbers, loop numbers, areas, and perimeters, and cell covered area.

### 2.7. Statistical Analysis

All the results reported represent average values from three independent experiments. All error estimates are given as the mean ± standard error of the mean (S.E.M). Statistical differences were determined using one-way analysis of variance. The *p*-values of less than 0.05 were considered significant.

## 3. Results

### 3.1. RHOG Positively Regulates Tube Formation in ECV Cells

**Figure 1:**
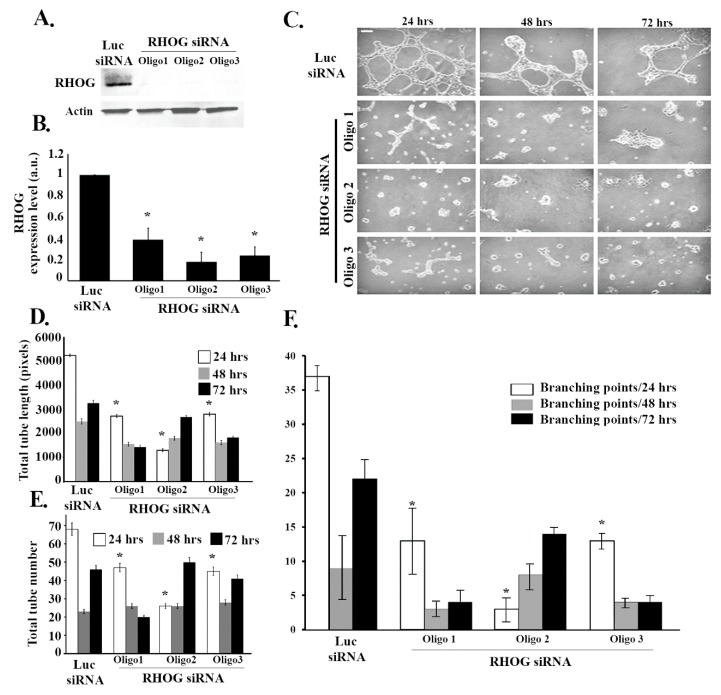
RHOG positively regulates tube formation in ECV cells. ECV cells were transfected with luciferase control siRNA or with RHOG siRNA. Three different siRNA oligos against RHOG were used in each experiment. (**A**) The cells were lysed and immunoblotted using Western blot analysis for RHOG (upper gel) or for actin (lower gel) for the loading control. (**B**) Western blot bands were quantified using imageJ and normalized to the number of total proteins and expressed as fold decreases from the luciferase control. Data are the mean ± SEM of three independent experiments. * *p* < 0.05 indicates statistically significant differences. (**C**) Representative images of the tube formation assay on the growth factor-reduced Matrigel by ECV at 24, 48, and 72 h after plating. (**D**–**F**) Quantitation of (C) for the total tube length, total tube number, and the number of branching points, respectively. Data are the mean ± SEM of three independent experiments. * *p* < 0.05 indicates statistically significant differences with the luciferase control. The scale bar is 100 μm.

To investigate the role of RHOG in angiogenesis, RHOG was knocked down using small interfering siRNA in ECV cells and a tube formation assay was performed. The efficiency of the knockdown was determined using Western blot (Figure 1A). The 3 different siRNA used reduced RHOG expression by 60–80% in the transfected cells compared to Luciferase siRNA-transfected control cells (β−actin was used as loading control) (Figure 1A,B). Then, the effect of RHOG knockdown on angiogenesis was determined by testing the effect of RHOG knockdown in a tube formation assay. The Wimtube Software was used to analyze the total tube length, the total number of tubes, and branching points before and after the knockdowns. Our results showed a significant decrease (approximately 50%) in the total tube length in response to the RHOG knockdown at 24, 48, and 72 h post-plating (Figure 1C,D). The number of tubes in addition to the number of branching points at each of the three time intervals also showed a decrease (Figure 1C,E,F). This showed that the formation of blood vessels in vascular endothelial cells, as reflected in the tube formation assay, is RHOG-dependent.

### 3.2. RAC1 Positively Regulates Tube Formation in ECV Cells

**Figure 2:**
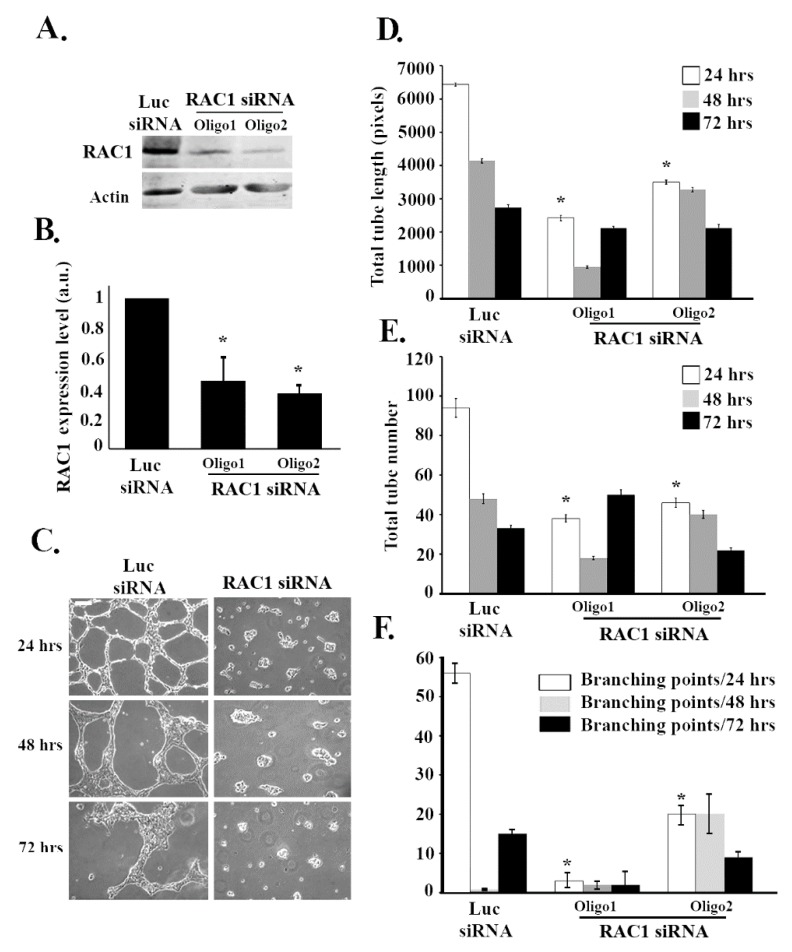
RAC1 positively regulates tube formation in ECV cells. ECV cells were transfected with the luciferase control siRNA or with RAC1 siRNA. Two different siRNA oligos against RAC1 were used in each experiment. (**A**) The cells were lysed and immunoblotted using Western blot analysis for RAC1 (upper gel) or for actin (lower gel) for the loading control. (**B**) Western blot bands were quantified using imageJ and normalized to the number of total proteins and expressed as fold decreases from the luciferase control. Data are the mean ± SEM of three independent experiments. * *p* < 0.05 indicates statistically significant differences. (**C**) Representative images of the tube formation assay on the growth factor-reduced Matrigel by ECV after 24, 48, and 72 h after plating. (**D**–**F**) Quantitation of (C) for the total tube length, total tube number, and the number of branching points, respectively. Data are the mean ± SEM of three independent experiments. * *p* < 0.05 indicates statistically significant differences with the luciferase control. The scale bar is 100 μm.

**Figure 3:**
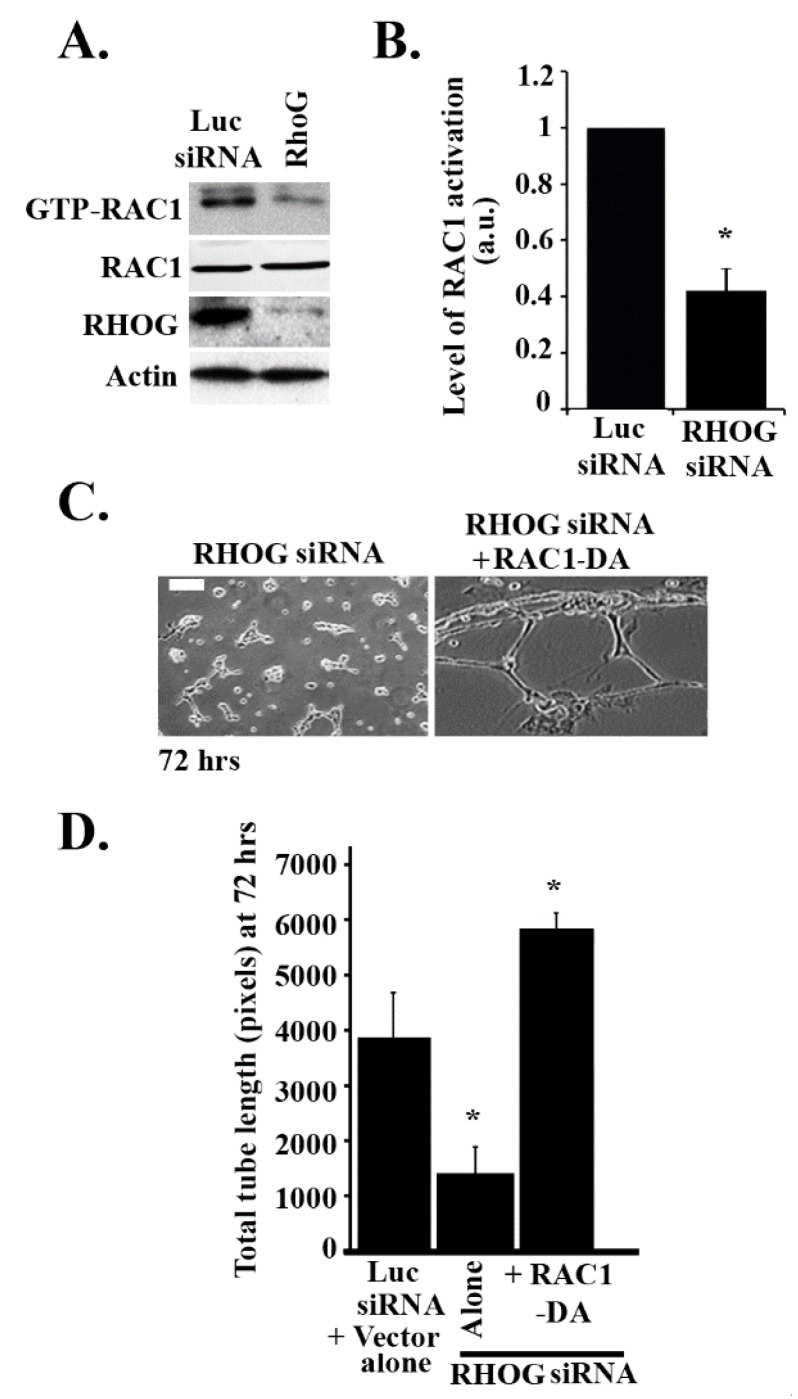
RHOG activates RAC1 leading to tube formation in ECV cells. (**A**) Cells were transfected with either luciferase or RHOG siRNA. Cells were then lysed and incubated with GST-CRIB (CDC42 and RAC interactive binding domain) to pull down the active RAC1. Samples from the pull-down as well as the total lysates were blotted against RAC1. The lower 2 gels are Western blots for RHOG for the knockdown control and actin for the loading control. (**B**) Quantitation of GTP-RAC1 from (A) normalized to total RAC1 and expressed as a fold decrease from the luciferase control. Data are the mean ± SEM of three independent experiments. * *p* < 0.05 indicates statistically significant differences. (**C**) Representative images of the tube formation assay (72 h) of ECV cells treated with RHOG siRNA (left) or RHOG siRNA/RAC1-DA (right). The scale bar is 100 μm. (**D**) Quantitation of (C) for the total tube length with the luciferase siRNA/vector alone as a control. The data are the mean ± SEM of three independent experiments. * *p* < 0.05 indicates statistically significant differences.

Since RHOG has been found in many systems to be an upstream regulator of RAC1 [33], it was interesting to examine if RAC1 also regulates tube formation in ECV cells. RAC1 was knocked down using 2 different siRNA oligos. The Western blot confirmed that RAC1 targeting siRNA significantly reduced the protein levels of RAC1 (Figure 2A,B). As expected, RAC1 knockdown resulted in a significant decrease in the total tube length and the total number of tubes at 24, 48, and 72 h (Figure 2C–E). Moreover, the number of branching points also decreased upon knockdown due to the decrease in the number of tube formations (Figure 2C,F). In order to determine if RHOG directly regulates RAC1 in these cells, RHOG was knocked down, and RAC1 activation was tested using a pull-down assay. In brief, cells were lysed and incubated with GST-CRIB (Cdc42 and Rac interactive binding domain from PAK1) for 30 min at 4 °C. Active RAC1 was then detected by Western blot. Indeed, in cells transfected with RHOG siRNA, the level of active RAC1 substantially decreased (Figure 3A,B). Furthermore, RHOG siRNA-transfected ECV cells were able to reverse the RHOG siRNA-mediated tube formation inhibition when co-transfected with a dominant active RAC1 construct (RAC1-Q61L) (Figure 3C,D).

### 3.3. RHOG Activates RAC1 through the Activation of CDC42 in ECV Cells

**Figure 4:**
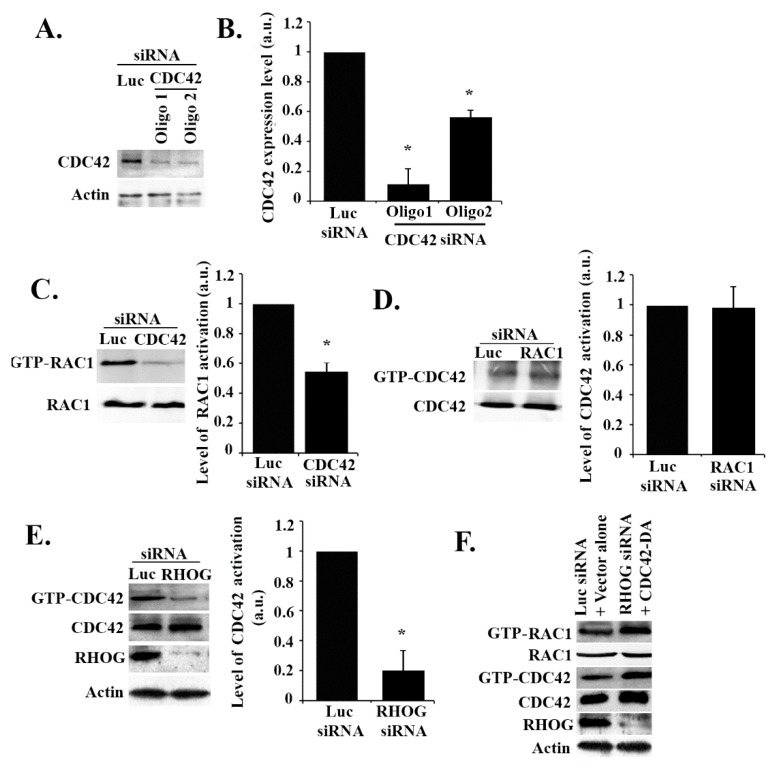
RHOG activates CDC42 which activates RAC1 in ECV cells. (**A**) Cells were transfected with either the luciferase control or CDC42 siRNA (2 oligos). The cells were then lysed, and the samples blotted with anti-CDC42 antibody. (**B**) Quantitation of (A) (knockdown efficiency) normalized to actin and expressed as a fold decrease compared to the luciferase control. Data are the mean ± SEM of three independent experiments. * *p* < 0.05 indicates statistically significant differences. (**C**) Cells were transfected with either luciferase or CDC42 siRNA (as in A), lysed, and incubated with GST-CRIB to pull down active RAC1. Samples, as well as total lysates (lower gel), were then blotted with RAC1 antibody. The graph is a quantitation of the gel to the total RAC1 and is expressed as a fold decrease compared to the luciferase control. The data are the mean ± SEM of three independent experiments. * *p* < 0.05 indicates statistically significant differences. (**D**) Cells were transfected with either luciferase or RAC1 siRNA lysed and incubated with GST-CRIB to pull down active CDC42. Samples, as well as total lysates (lower gel), were then blotted with the CDC42 antibody. The graph is a quantitation of the gel to total CDC42 and expressed as a fold decrease compared to the luciferase control. Data are the mean ± SEM of three independent experiments. * *p* < 0.05 indicates statistically significant differences. (**E**) Cells were transfected with either luciferase or RHOG siRNA lysed and incubated with GST-CRIB to pull down active CDC42. Samples were then blotted with anti-CDC42 and total lysates with anti-CDC42, anti-RHOG (for knockdown efficiency), and anti-actin (for loading control). The graph is a quantitation of GTP-CDC42 ratioed to total CDC42 and expressed as fold decrease compared to luciferase control. Data are the mean ± SEM of three independent experiments. * *p* < 0.05 indicates statistically significant differences. (**F**) Cells were transfected with either luciferase control and vector alone or with RHOG siRNA and CDC42-DA. Then, cells were lysed and incubated with GST-CRIB (CDC42 and RAC1 interactive binding domain) (GTP-RAC1 and GTP-CDC42 gels) to pull down active CDC42 and RAC1. Samples were then blotted with RAC1 and CDC42 antibodies. Total cell lysates were also blotted with RAC1, CDC42 antibodies. The lower 2 gels are Western blots for RHOG and actin for knockdown efficiency.

To test if CDC42 activates RAC1 in these cells, CDC42 was knocked down, and the effect on RAC1 activation was tested. As seen in Figure 4A,B, the 2 CDC42 siRNA oligos used showed high efficiency of suppression of CDC42 expression. The CDC42 knockdown led to a similar effect to that of the RHOG knockdown on RAC1 activation, as seen by the pull-down assay, with more than a 50% decrease in activation in the CDC42-depleted cells (Figure 4C). To test if the reverse is also true, CDC42 activation was examined in RAC1 siRNA-transfected cells, but the RAC1 knockdown showed no effect, confirming that CDC42 is upstream from RAC1 in these cells (Figure 4D). Similarly, in order to test if CDC42 is also downstream from RHOG, ECV cells were transfected with RHOG siRNA, which led to an approximately 80% decrease in CDC42 activation as compared to the luciferase-transfected cells (Figure 4E). In addition, when ECV cells were co-transfected with RHOG siRNA and a dominant active CDC42 construct (CDC42-Q61L), this recovered the RHOG knockdown-mediated decrease in RAC1 activation seen in cells transfected with RHOG siRNA alone (Figure 4F). This data showed CDC42/RAC1 as a signaling module downstream from RHOG in EVC cells, with CDC42 activity seeming necessary and sufficient in mediating the RHOG activation of RAC1. We also looked at a potential activation of RHOA downstream from RHOG. When either RHOG or CDC42 were knocked down, the level of activation of RHOA was not affected in these cells (Supplemental Figure S1), suggesting RhoA operates in a separate pathway.

### 3.4. RHOG Stimulates Tube Formation through CDC42 and RAC1

**Figure 5:**
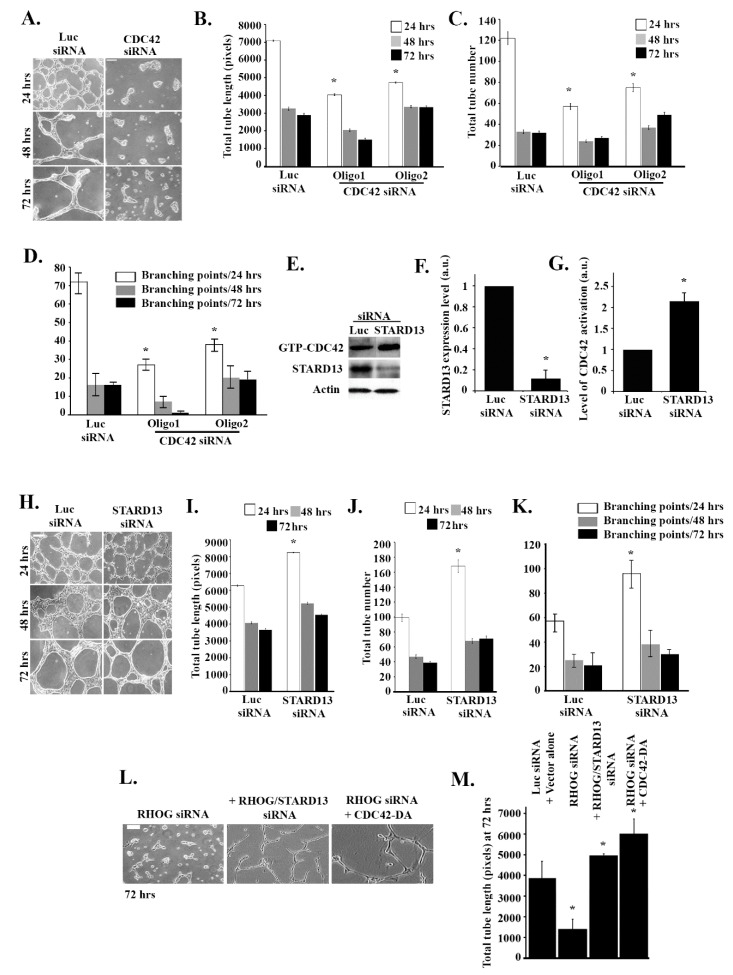
RHOG stimulates tube formation through CDC42 and RAC1. ECV-cells were transfected with the luciferase control, CDC42 (2 oligos) or STARD13 siRNA and plated on growth factor-reduced Matrigel by ECV for 24, 48, or 72 h. (**A**) Representative images of tube formation assay for the luciferase control and the CDC42 knockdown cells at 24, 48, and 72 h after plating. The scale bar is 100 μm. (**B**–**D**) Quantitation of (A) for the total tube length, total tube number, and the number of branching points, respectively. Data are the mean ± SEM of three independent experiments. * *p* < 0.05 indicates statistically significant differences with the luciferase control. (**E**) Cells were transfected with either luciferase or STARD13 siRNA, and cells were then lysed and incubated with GST-CRIB to pull down active CDC42. The samples were then blotted with CDC42 antibody. Total cell lysates were also blotted with STARD13 for knockdown efficiency and actin for the loading control. (**F**) Quantitation of STARD13 expression (cells −/+ STARD13 siRNA) in the Western blot bands from (E) normalized to actin (lowest gel) and expressed as a fold change. Data are the mean ± SEM of three independent experiments. * *p* < 0.05 indicates statistically significant differences. (**G**) Quantitation of GTP-CDC42 from (E) normalized to total CDC42 and expressed as a fold change from the luciferase control. Data are the mean ± SEM of three independent experiments. * *p* < 0.05 indicates statistically significant differences. (**H**) Representative images of the tube formation assay for the luciferase control and the STARD13 knockdown cells at 24, 48, and 72 h after plating. The scale bar is 100 μm. (**I**–**K**) Quantitation of (H) for the total tube length, total tube number, and the number of branching points, respectively. Data are the mean ± SEM of three independent experiments. * *p* < 0.05 indicates statistically significant differences with the luciferase control. **(L)** Representative images of tube formation assay (72 h) of ECV cells treated with RHOG siRNA (left), RHOG/STARD13 siRNA (double knockdown) (middle), or RHOG siRNA/CDC42-DA (left). The scale bar is 100 μm. (**M**) Quantitation of (L) for total tube length with the luciferase siRNA/vector alone as the control. Data are the mean ± SEM of three independent experiments. * *p* < 0.05 indicates statistically significant differences.

To directly confirm that CDC42 is required for tube formation, CDC42 was knocked down using the oligos described in Figure 4. As expected, the CDC42 knockdown inhibited tube formation relative to non-treated cells (Figure 5A). Quantitative analysis confirmed that the length and numbers of the tubes, as well as branching point numbers, decreased in response to the CDC42 knockdown (Figure 5B–D). We then looked at the effect of knocking down the CDC42/RHOA-GAP STARD13 on tube formation in these cells. The efficiency of the knockdown in these cells was tested by Western blot (Figure 5E,F). When STARD13 was knocked down, this led to an increase in CDC42 activation, as expected (Figure 5E,G). Surprisingly, STARD13 does not seem to affect RhoA activation in these cells (Supplemental Figure S1). STARD13 knockdown led to an increase in tube formation and in the totals of the length and numbers of the tubes, as well as in the branching point numbers (Figure 5H–K). Finally, when dominant active CDC42 (CDC42-Q61L) or STARD13 siRNA were added to RHOG siRNA, this rescued the tube inhibition (Figure 5L,M). These results confirmed that RHOG regulates tube formation through the CDC42/RAC1 pathway

### 3.5. RHOG/CDC42/RAC1 Leads to Tube Formation through the ERK Pathway

**Figure 6:**
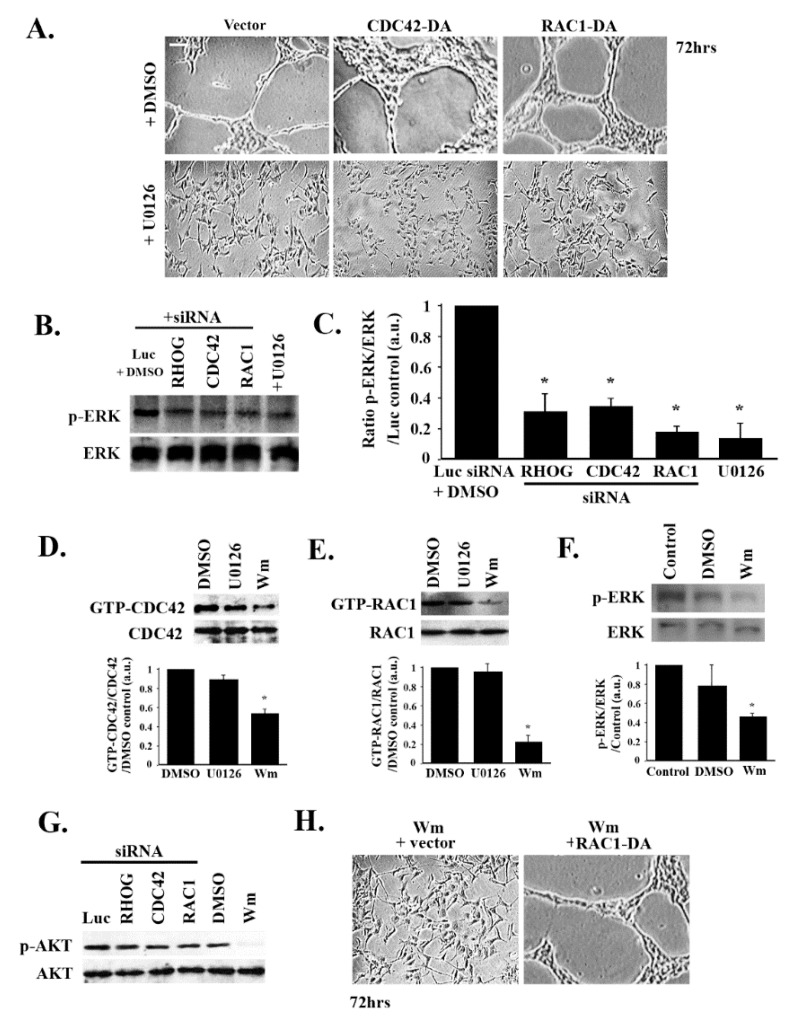
RHOG/CDC42/RAC1 leads to tube formation through ERK and downstream from PI3K. (**A**) Representative images of a tube formation assay (72 h after plating) of ECV cells transfected with the vector alone, CDC42-DA, or RAC1-DA, and treated with DMSO or with 10 μM U0126 (24 h before imaging). The scale bar is 100 μm. (**B**,**C**) ECV cells were transfected either with luciferase, RHOG, CDC42, or RAC1 siRNA, or transfected with the vector alone, CDC42-DA, or RAC1-DA, and treated with 10 μM U0126 (24 h) or with the carrier (DMSO). (**B**) Western blot of the lysates from the different conditions mentioned above against p-ERK and ERK as controls. (**C**) Quantitation of (B) normalized to ERK and expressed as a fold difference from the luciferase control. Data are the mean ± SEM of three independent experiments. * *p* < 0.05 indicates statistically significant differences. (**D**) ECV cells were treated with DMSO or 10 μM U0126 or 100 nM Wortmannin (2 h) or left untreated. Cells were then lysed and pull-down assays for CDC42 were performed. Total lysates were also blotted for CDC42 as the control. The graph is a quantitation of the gel normalized to total CDC42 and expressed as a fold change to the DMSO control. Data are the mean ± SEM of three independent experiments. * *p* < 0.05 indicates statistically significant differences. (**E**) ECV cells were treated with DMSO, 10 μM U0126, or 100 nM Wortmannin (2 h), or left untreated. Cells were then lysed and pull-down assays for RAC1 were performed. Total lysates were also blotted for RAC1 as a control. The graph is a quantitation of the gel normalized to total RAC1 and expressed as a fold change to the DMSO control. Data are the mean ± SEM of three independent experiments. * *p* < 0.05 indicates statistically significant differences. (**F**) Cells were treated with DMSO control, Wortmannin, or left untreated. Cells were then lysed and blotted against p-ERK and ERK for control. The graph is a quantitation of the gel normalized to ERK and expressed as fold change to the control. Data are the mean ± SEM of three independent experiments. * *p* < 0.05 indicates statistically significant differences. (**G**) Cells were treated with DMSO or 100 nM Wortmannin (2 h) or were transfected with luciferase, RHOG, CDC42, or RAC1 siRNA (72 h). Cells were then lysed and blotted against p-AKT and AKT for the control. (**H**) Representative images of a tube formation assay (72 h after plating) of ECV cells transfected with the vector alone or with CDC42-DA and treated with 100 nM Wortmannin (2 h).

The role of MAPK pathways (particularly the MEK/ERK pathway) in the regulation of vascular development and angiogenesis has been established [35]. Accordingly, the effect of ERK inhibition on tube formation in these cells was examined. For this treatment, the cells were plated for 48 h then treated with 10 μM of the specific MEK1/2 inhibitor U0126 for 24 h and then imaged. The U0126-treated cells showed inhibition of tube formation (Figure 6A). Surprisingly, when the U0126-treated ECV cells were transfected with CDC42-DA or RAC1-DA, the cells were not able to rescue the U0126-dependent inhibition of tube formation compared to control cells (Figure 6A). In addition, Western blot analysis showed a decrease in p-ERK when the cells were treated with U0126 or with RHOG, RAC1, or CDC42 siRNA (Figure 6B,C). The reverse experiment was also performed. When ECV cells were treated with U0126, this did not affect CDC42 or RAC1 activation (Figure 5D,E). This, collectively, suggested that RHO GTPases act upstream from the MAPK pathway, leading to tube formation in these cells.

### 3.6. RHOG/CDC42/RAC1 Leads to Tube Formation Downstream from the PI3K Pathway

Contrary to ERK inhibition, treating ECV cells with 100 nM of the specific PI3K inhibitor Wortmannin led to a decrease in the activation of CDC42 and RAC1, as seen by the pull-down assay (Figure 6D,E). Cells treated with Wortmannin also showed a decrease in p-ERK (Figure 6F). In addition, knocking down RHOG, CDC42, or RAC1 did not affect PI3K signaling in these cells, as seen by the p-AKT Western blot (Figure 6G). The role of PI3K in tube formation in these cells was next examined. Wortmannin treatment led to a complete loss of tube formation (Figure 6H). However, treating RAC1-DA-transfected cells with the PI3K inhibitor recovered the tube formation (Figure 6H). Collectively, this data suggested that PI3K is upstream from the MAPK pathway and RHO GTPases in these cells, leading to tube formation.

### 3.7. RHOA/ROCK1/ROCK2 Regulate Tube Formation in an ERK-Independent Manner

**Figure 7:**
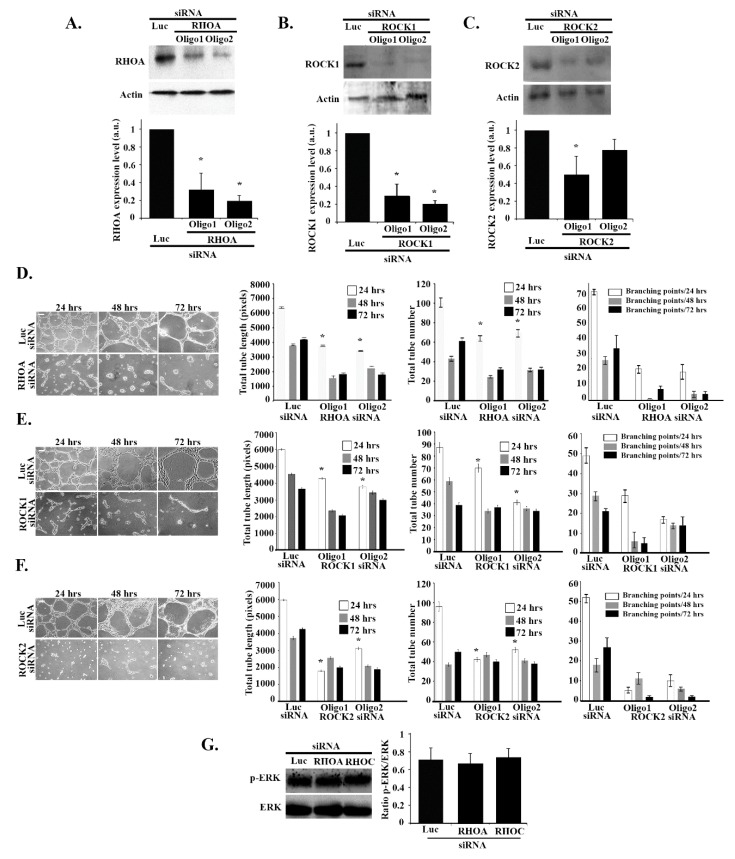
RHO/ROCK regulate tube formation in an ERK-independent manner. (**A**–**C**) ECV cells were transfected with the luciferase control siRNA or with two different RHOA, ROCK 1 and 2 siRNA oligos, respectively. The cells were lysed and immunoblotted by Western blot analysis with anti-RHOA, anti-RHOC, anti-ROCK1, and anti-ROCK2 antibodies, and with anti-actin as the control. The graphs are quantitation of the gels normalized to actin and expressed as fold decreases compared to the luciferase control. Data are the mean ± SEM of three independent experiments. * *p* < 0.05 indicates statistically significant differences. (**D**–**F**) Representative images of ECV tube formation assay (Scale bar is 100 μm) and quantitation of total tube length, total tube number, and the number of branching points, respectively, after 24, 48, and 72 h of treatment with RHOA, ROCK 1 and 2 siRNA vs. control. Data are the mean ± SEM of three independent experiments. * *p* < 0.05 indicates statistically significant differences with the luciferase control. (**G**) Cells were transfected with luciferase, RHOA, or RHOC siRNA, lysed and blotted against p-ERK and ERK for control. The gels were quantitated, and the level of ERK phosphorylation was expressed as the ratio p-ERK/ERK. Data are the mean ± SEM of three independent experiments. * *p* < 0.05 indicates statistically significant differences.

Finally, we aimed to investigate the role of other RHO GTPases such as RHOA, RHOC, and their downstream effectors, ROCK 1 and 2, in tube formation. RHOA, RHOC, ROCK1, and ROCK2 were knocked down in ECV cells (2 oligos were used for each) and the knockdown efficiencies were tested for by Western blot and quantitation (Figure 7A–C and Figure S2A). As Figure 7 shows, the total tube length and numbers and the number of branching points decreased when the cells were treated by RHOA and ROCK1 and 2 (Figure 7D–F) but not RHOC (Figure S2B and S2C). Interestingly, Western blot analysis revealed that the level of expression of p-ERK was not affected by RHOA or RHOC siRNA treatment (Figure 7G). These data suggest that, as opposed to the RHOG-CDC42-RAC1 axis of GTPases, the RHOA/ROCK1/2 branch regulates tube formation in ECV cells in an ERK-independent manner.

## 4. Discussion

During angiogenesis, endothelial cells build a network of new vessels to meet the oxygen and nutrient needs of the growing primary tumor, as well as to provide a route for metastasis, rendering the tumor increasingly malignant [5,36]. Understanding angiogenesis and the signaling pathway that regulate the process is instrumental in anti-angiogenic cancer therapy research. RHOG, a member of the RHO family of GTPases, is a main regulator of the actin cytoskeleton, hence it is expected to play a central role in regulating endothelial cell tube formation during angiogenesis [26,33]. The role of the RHO GTPases RAC1, CDC42, RHOA, and RHOC during angiogenesis has been extensively studied [2,8,23,24]. RHOG has been also established as a regulator of angiogenesis and a necessary component of a signaling cascade (along with CDC42 and RAC1), leading to filopodia formation and subsequent tubular remodeling [37]. In this study, we further examine the cross-talk between RHOG and other members of the RHO family of GTPases, particularly CDC42, RAC1, RHOA, and RHOC in the vascular endothelial cells. We also examine the upstream regulators of RHOG in a tube formation assay.

To investigate the role of RHOG during angiogenesis, the effect of knocking down RHOG in a tube formation assay was examined in vascular endothelial cells (ECV cells). We find the formation of blood vessels in these cells to be RHOG-dependent. Testing the effect of RAC1 and CDC42 knockdowns reveal that both CDC42 and RAC1 are also required for tube formation in these cells. This is consistent with several reports that previously established the role of CDC42 and RAC1 in angiogenesis [9,23,38]. When dominant active CDC42 or RAC1 constructs are expressed in the RHOG siRNA-transfected cells, the cells regain their ability to form tubes, showing that RHOG regulates tube formation through the CDC42/RAC1 pathway and that RHOG and CDC42/RAC1 are in the same pathway, rather than signal in parallel. This is further directly shown through the direct inhibition of the RHOG siRNA of CDC42 and RAC1 activation in a pull-down assay. This study also shows that CDC42 is upstream from RAC1 in these cells. While knocking down RAC1 did not have an effect on CDC42 activation, the reverse was not true. In addition, in cells co-transfected with RHOG siRNA along with the dominant active CDC42 construct RAC1 activation was recovered (consistent with the tube formation recovery). In fact, in these samples, RAC1 activation appeared to be slightly higher than the control, potentially due to the constitutive exaggerated activity of CDC42-DA (as confirmed by the CDC42 pull-down assay). CDC42 has been indeed previously reported to be upstream from RAC1 in many cell types [39,40]. In addition to the CDC42 dominant active construct, we also used siRNA against the known CDC42/RHOA GAP STARD13 to further manipulate the level of CDC42 activation in these cells [41]. Whereas in other cell types STARD13 is a GAP for CDC42 and RHOA [42,43,44], in vascular endothelial cells it appears to only inhibit CDC42. An increase in RAC1 activation, compared to control, is also seen in cells transfected with STARD13 siRNA, potentially due to the increase in CDC42 activation described above. Consistently, this is also reflected in the increase in tube formation compared to control cells.

**Figure 8:**
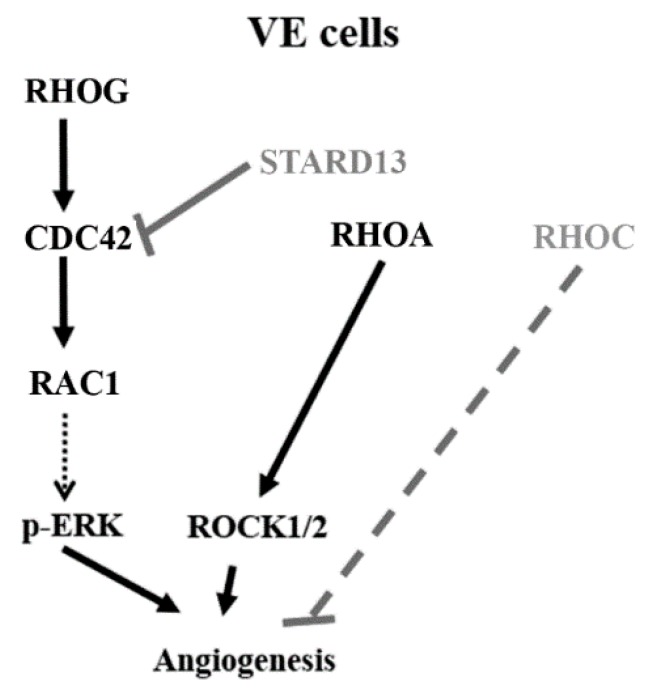
Suggested model based on the data presented in this study: RHOG positively regulates tube formation assay in ECV cells through a CDC42-RAC1-ERK. PI3K appears to be an upstream regulator of RHO GTPases leading to tube formation. RHOA and C seem to have different roles in tube formation. While RHOA leads to positive regulation of the process through ROCK1/2 as downstream effectors, RHOC does not seem to be necessary for tube formation. Finally, RHOA, unlike the RHOG-CDC42-RAC1 pathway, does not seem to exert its effect on tube formation through the MAPK pathway.

Collectively, this data shows that RHOG activates CDC42, which in turn activates RAC1, in this pathway (Figure 8-model). Our data are consistent with studies that place RHOG upstream from CDC42/RAC1 in other cells [28,31,33,37,45]. In fact, an earlier study found RHOG to signal upstream from RAC1/CDC42 in endothelial cells leading to filopodia formation essential for tube remodeling [37]. Other studies suggest that RHOG signals in parallel to RAC1 and CDC42 [27]. In fact, RHOG was found in those studies to be activated by RAC1-specific GEFs, such as VAV2, VAV3, and TRIO [46,47], suggesting RHOG acts in parallel to RAC1. This contradiction in reporting could be simply due to different cell types and systems, as has been repeatedly seen for RHO GTPases [48].

The role of the MEK/ERK pathway in the regulation of vascular development and angiogenesis is established [35]. Our data confirm this role through the inhibition of tube formation following treatment of ECV cells with the specific MEK inhibitor U0126. While classically MAPKs are thought to regulate RHO GTPases [49], ERK seems to act downstream from the RHOG/CDC42/RAC1 pathway in these cells. Indeed, knocking down RHOG, CDC42 or RAC1 leads to a decrease in the level of ERK phosphorylation, comparable to inhibition with U0126 (U0126 treatment did not affect CDC42 or RAC1 activation). In a previous study from our laboratory, we observed an increase in ERK phosphorylation in response to STARD13 knockdown in astrocytoma cells, also placing ERK downstream from CDC42 [42]. In addition, overexpressing dominant active CDC42 or RAC1 did not rescue the U0126-mediated inhibition of tube formation, again placing ERK downstream from the RHOG/CDC42/RAC1 pathway in these cells.

Another important regulator of angiogenesis and of RHO GTPases is phosphatidylinositol-3-kinase (PI3K) [16,39,50]. Tube formation is PI3K-dependent in these cells. However, contrary to the effect on ERK, knocking down RHOG, CDC42, or RAC1 does not affect PI3K signaling in these cells. This suggests that, contrary to MAPK, RHO GTPases are not upstream of PI3K in this pathway. Indeed, cells treated with Wortmannin show a decrease in p-ERK and in the activation of CDC42 and RAC1 by pull-down assay, placing PI3K upstream of the RHOG-CDC42-RAC1-ERK axis during tube formation in these cells. Consistently, treating RAC1-DA-transfected cells with the PI3K inhibitor recovered the tube formation. This suggested that, similarly to RHOG, RAC1 activation is downstream from PI3K and directly activating tube formation abrogating the need for PI3K signaling.

Finally, RHOA, RHOC and their downstream effectors ROCK1 and 2 have been established as regulators of angiogenesis [2,8,10,25]. Our data confirm the positive role of RHOA/ROCK1/ROCK2 in tube formation. Surprisingly, RHOC shows a negative effect on tube formation since the knockdown of RHOC led to a notable increase in tube formation. This is in accordance with previous reports which described antagonistic roles of the 2 isoforms RHOA and RHOC, albeit during cancer cell invasion [14]. The effect of RHOA/ROCK1/ROCK2 does not seem to be mediated through ERK. In addition, RHOG knockdown, CDC42 knockdown, and STARD13 knockdown did not have any effect on RHOA activation. This together suggests that, during tube formation, the RHOG-CDC42-RAC1 pathway and the RHOA-ROCK1/2 pathway operate in parallel and do not compensate for one another. It also suggests that, as opposed to the RHOG-CDC42-RAC1 axis of GTPases, the RHOA/ROCK1/2 branch regulates tube formation in ECV cells in an ERK-independent manner (Figure 8).

In conclusion, the data in this paper describes, for the first time, the role of RHOG signaling during angiogenesis in vascular endothelial cells. It also dissects its cross-talk with other RHO GTPases and regulation by PI3K. While the mechanism of activation of CDC42/RAC1 by RHOG, the MAPK by RAC1, as well as the intersection with the RHOA/ROCK1/2 pathways remain topics for future work, we believe this study sheds the light on RHOG as a potentially promising target for anti-angiogenesis in cancer therapeutics.

## Supplementary Materials

The following are available online at https://www.mdpi.com/2073-4409/8/2/171/s1, Figure S1: STARD13/CDC42 and RHOG do not regulate RHOA in ECV cells, Figure S2: Tube formation is not RHOC-dependent.
