# Supplementary material for: RHOG Activates RAC1 through CDC42 Leading to Tube Formation in Vascular Endothelial Cells
Source: Cells. 2019 Feb 18;8(2):171. doi: 10.3390/cells8020171 (PMC6406863; doi:10.3390/cells8020171)
Supplement: Supplementary file 1 [file cells-08-00171-s001.pdf]

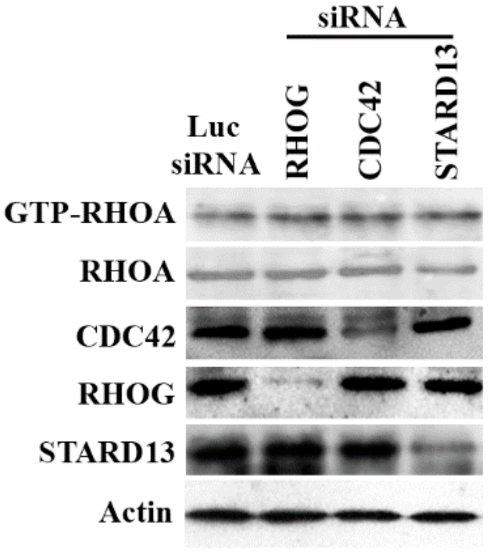

Figure S1. STARD13/CDC42 and RHOG do not regulate RHOA in ECV cells. Cells were transfected with either luciferase, RHOG, CDC42 or STARD13 siRNA, lysed and incubated with GST-RBD (Rhotekin-binding domain) to pull down active RHOA. Samples were then blotted with RHOA antibody and total lysates blotted with RHOA antibody (for total RHOA), CDC42, RHOG and STARD13 antibodies (for knock down efficiency) and actin for loading control.

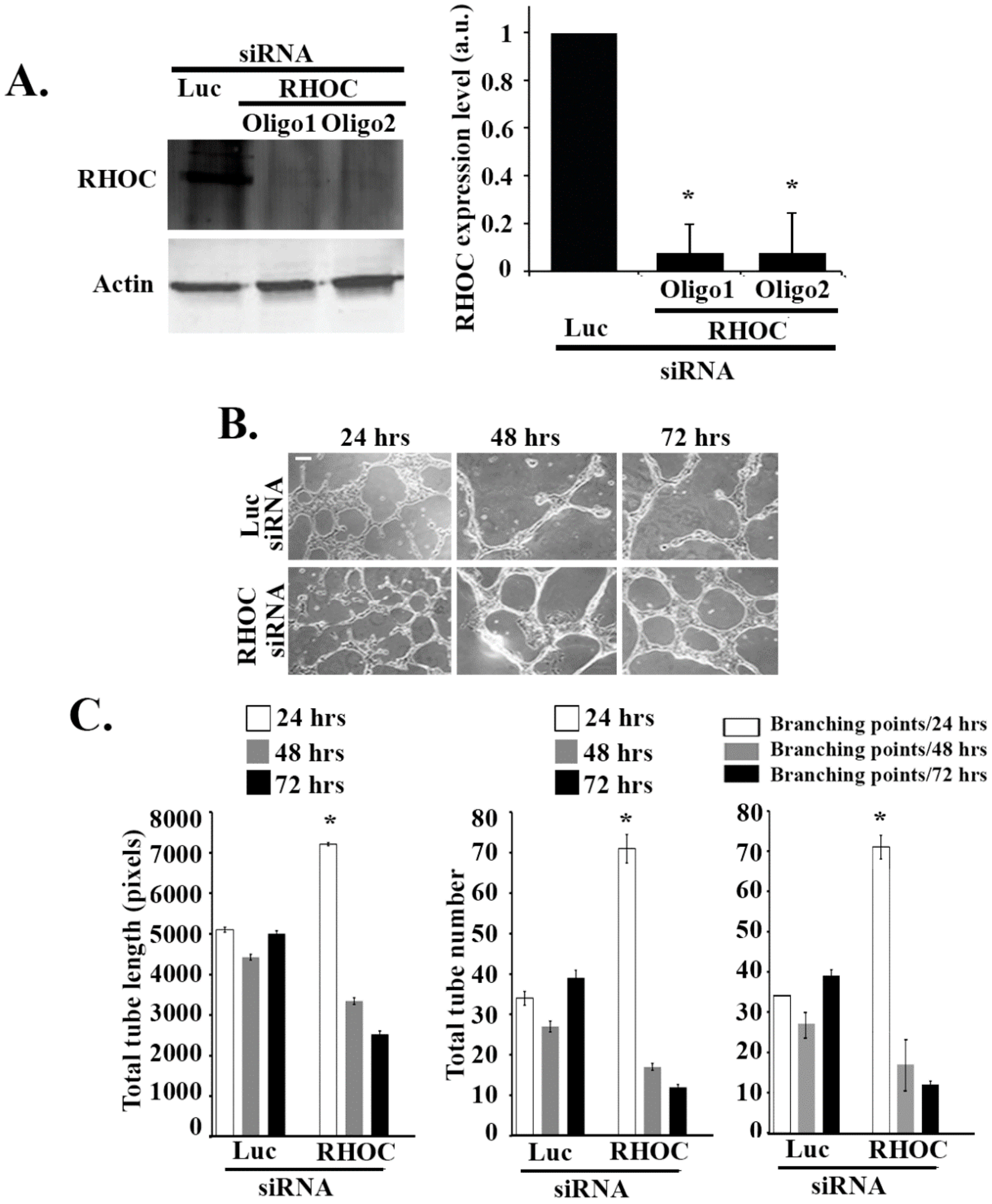

Figure 2. Tube formation is not RHOC-dependent. (A) ECV cells were transfected with luciferase control siRNA or with two different RHOC siRNA oligos. The cells were lysed and immunoblotted by western blot analysis with anti-RHOC and anti-actin as control. The graph is a quantitation of the gels normalized to actin and expressed as fold decrease compared to luciferase control. Data are the mean ± SEM of three independent experiments. *P<0.05 indicates statistically significant differences. **(B)** Representative images of ECV tube formation assay -/+ RHOC siRNA (Scale bar is 100 µm). **(C)** Quantitation of total tube length, total tube number, and number of branching points, respectively, after 24, 48, and 72 hrs of treatment with RHOC siRNA vs control. Data are the mean ± SEM of three independent experiments. *P<0.05 indicates statistically significant differences with luciferase control.
